# Chemotherapy-induced executioner caspase activation increases breast cancer malignancy through epigenetic de-repression of *CDH12*

**DOI:** 10.1038/s41389-023-00479-x

**Published:** 2023-06-24

**Authors:** Yuxing Wang, Ru Wang, Xiaohe Liu, Menghao Liu, Lili Sun, Xiaohua Pan, Huili Hu, Baichun Jiang, Yongxin Zou, Qiao Liu, Yaoqin Gong, Molin Wang, Gongping Sun

**Affiliations:** 1 Key Laboratory of Experimental Teratology, Ministry of Education, Institute of Molecular Medicine and Genetics, School of Basic Medical Sciences, Cheeloo College of Medicine, Shandong University, Jinan, Shandong 250012 China; 2 Key Laboratory of Experimental Teratology, Ministry of Education, Department of Histology and Embryology, School of Basic Medical Sciences, Cheeloo College of Medicine, Shandong University, Jinan, Shandong 250012 China; 3 Department of Breast and Thyroid Surgery, Shandong Provincial Hospital Affiliated to Shandong First Medical University, Jinan, Shandong 250021 China; 4 Department of Systems Biomedicine and Research Center of Stem Cell and Regenerative Medicine, School of Basic Medical Sciences, Cheeloo College of Medicine, Shandong University, Jinan, Shandong 250012 China

**Keywords:** Breast cancer, Metastasis

## Abstract

Cancer relapse and metastasis are major obstacles for effective treatment. One important mechanism to eliminate cancer cells is to induce apoptosis. Activation of executioner caspases is the key step in apoptosis and was considered “a point of no return”. However, in recent years, accumulating evidence has demonstrated that cells can survive executioner caspase activation in response to apoptotic stimuli through a process named anastasis. Here we show that breast cancer cells that have survived through anastasis (anastatic cells) after exposure to chemotherapeutic drugs acquire enhanced proliferation and migration. Mechanistically, cadherin 12 (CDH12) is persistently upregulated in anastatic cells and promotes breast cancer malignancy via activation of ERK and CREB. Moreover, we demonstrate that executioner caspase activation induced by chemotherapeutic drugs results in loss of DNA methylation and repressive histone modifications in the *CDH12* promoter region, leading to increased CDH12 expression. Our work unveils the mechanism underlying anastasis-induced enhancement in breast cancer malignancy, offering new therapeutic targets for preventing post-chemotherapy cancer relapse and metastasis.

## Introduction

Breast cancer is the most commonly diagnosed cancer and the leading cause of cancer death worldwide^1^. Although adjuvant chemotherapy and other targeted drugs significantly improved the overall survival in patients, post-therapeutic relapse and metastasis are still the major challenges to breast cancer treatment^2–4^. Induction of apoptosis is an important strategy to eliminate cancer cells^5–8^. A key step in apoptosis is executioner caspase activation, which results in cleavage of diverse protein substrates, leading to cell dismantlement^9–12^. In recent years, survival from executioner caspase activation after exposure to chemotherapeutic drugs has been reported in a group of cancer cell lines including breast cancer cells, cervical cancer cells, melanoma cells and ovarian cancer cells^13–15^. The process by which cells survive executioner caspase activation upon exposure to apoptotic stimuli was named anastasis^16–18^.

A couple of studies have demonstrated that anastasis can induce phenotypic changes in cancer cells. However, the features acquired by anastatic cells, which have survived stress-induced executioner caspase activation, vary among different types of cancer^13–15,19–21^. For example, anastatic cervical cancer cells exhibit enhanced migration and drug resistance^13^, whereas melanoma cells that survive from stress-induced executioner caspase activation only display elevation in migration but not in drug resistance^14^. Moreover, the mechanisms underlying anastasis-induced phenotypic changes are not clear. In this study, using a lineage tracing system to label cells that have experienced executioner caspase activation and their descendants, we demonstrate that anastasis induced by chemotherapeutic drugs renders breast cancer cells more proliferative and migratory, leading to enhanced in vivo cancer growth and metastasis. Moreover, we show that anastasis induces epigenetic de-repression of *CDH12*, the gene encoding cadherin 12 (CDH12), which in turn promotes proliferation and migration of breast cancer cells through activating ERK and CREB. Our work unveils anastasis and CDH12 as potential targets for preventing breast cancer relapse and metastasis following chemotherapy.

## Results

### Breast cancer cells undergo anastasis upon treatment of chemotherapeutic drugs

**Fig. 1:**
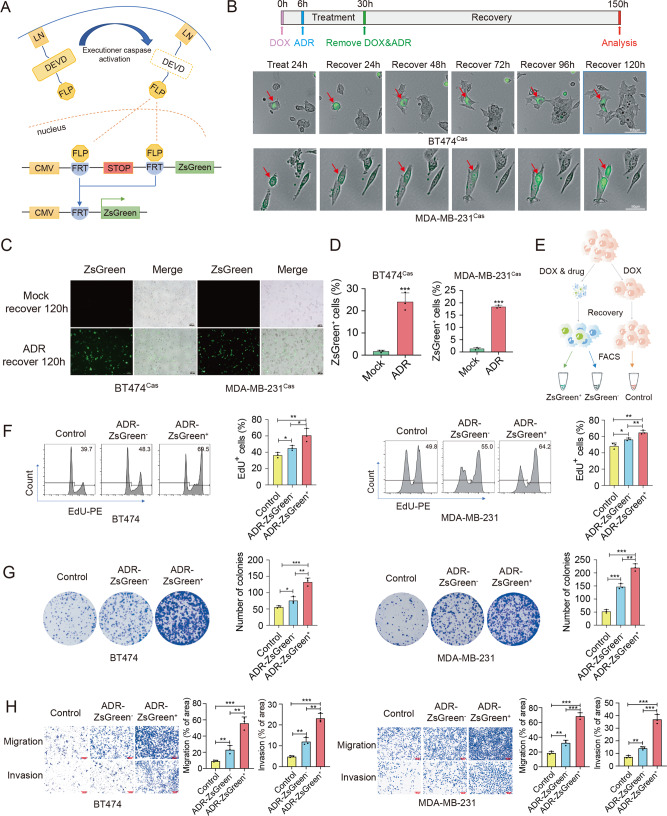
Breast cancer cells that survive ADR-induced executioner caspase activation acquire enhanced proliferation and migration. **A** Schematic of mCasExpress. LN: Lyn11-NES. **B** The representative images from time-lapse live imaging of BT474^Cas^ and MDA-MB-231^Cas^ cells during 120 h recovery after ADR treatment. The red arrows point to cells undergoing anastasis. Scale bar in upper row is 100 μm. Scale bar in lower row is 50 μm. **C**, **D** Images (**C**) and flow cytometry analysis (**D**) of BT474^Cas^ and MDA-MB-231^Cas^ cells after 120 h recovery from ADR or mock treatment. Scale bars in (**C**) are 200 μm. *n* = 3 in (**D**). **E** Schematic of the cell sorting process. **F**–**H** The results of EdU incorporation analysis (**F**), colony formation assays (**G**) and transwell assays (**H**) on the ADR-ZsGreen^+^, the ADR-ZsGreen^−^ and the control cell populations derived from BT474 and MDA-MB-231 cells. *n* = 3. Scale bars in (**H**) are 200 μm. Data are presented as mean ± SEM. Statistical significance was determined using one-way ANOVA with Tukey test for comparing three or more groups or *t*-test for comparing two groups. *P < 0.05; **P < 0.01; ***P < 0.001.

To investigate the role of anastasis in breast cancer relapse and metastasis after chemotherapy, we generated BT474 and MDA-MB-231 cells carrying mCasExpress, a lineage tracing system for cells that have experienced executioner caspase activation^15,22^. mCasExpress is comprised of two components, a DNA recombinase FLP that can be activated by active executioner caspases, and a FLP activity reporter *FRT-STOP-FRT-ZsGreen*. FLP is sequestered on membrane by fusing to Lyn11 sequence and a nuclear export signal (NES) through a short peptide containing executioner caspase-specific cleavage site, DEVD. When executioner caspases are activated, the DEVD site between FLP and Lyn11-NES (LN) sequence is cleaved, resulting in nuclear translocation of FLP, which in turn leads to removal of the transcriptional termination signal between the two FRT sites, and the subsequent expression of green fluorescent protein ZsGreen (Fig. 1A). Thus, with mCasExpress, cells that have experienced executioner caspase activation as well as their daughter cells will be labeled with green fluorescence (designated ZsGreen^+^ cells). Considering that some cultured cells may have the basal level of caspase activity or experience transient caspase activation during in vitro culture, the caspase-activatable FLP is placed downstream of a doxycycline (DOX)-inducible promoter to minimize the accumulation of ZsGreen^+^ cells during culture. We treated BT474 cells and MDA-MB-231 cells carrying mCasExpress (designated as BT474^Cas^ and MDA-MB-231^Cas^, respectively) with adriamycin (ADR) for 24 h, then replaced the drug-containing medium with fresh culture medium to allow the cells to recover. Live imaging demonstrated that at the end of ADR treatment, some shrunk BT474^Cas^ and MDA-MB-231^Cas^ cells emitted green fluorescence. During the course of 120 h recovery, a number of green shrunk cells gradually restored normal morphology and proliferated, indicating these cells survived after ADR-induced executioner caspase activation (Fig. 1B, red arrows). At 120 h after drug removal, the ADR-treated BT474^Cas^ and MDA-MB-231^Cas^ cells contained about 24 and 18% ZsGreen^+^ cells, respectively, while only about 2% were ZsGreen^+^ in cells recovered from mock treatment (Fig. 1C, D).

### Anastatic breast cancer cells acquire enhanced proliferation and migration

To investigate whether anastasis grants breast cancer cells any new features, we collected the ZsGreen^+^ cells (anastatic cells, ADR-ZsGreen^+^) and the ZsGreen^−^ cells (cells without executioner caspase activation, ADR-ZsGreen^−^) from BT474 and MDA-MB-231 cells recovered from ADR treatment by fluorescence-activating cell sorting (FACS). Cells recovered from mock treatment were also applied to FACS and the ZsGreen^−^ cells were collected as control populations (Fig. 1E). All the isolated populations can grow in vitro. The purity of these cell populations after long-term culture was verified by flow cytometry and genotyping (Fig. S1A, B).

We compared the in vitro proliferation, migration and invasion capacity among the ADR-ZsGreen^+^, the ADR-ZsGreen^−^ and the control populations. EdU incorporation assays and colony formation assays showed that the ADR-ZsGreen^+^ cells were more proliferative than the ADR-ZsGreen^−^ and the control cells (Fig. 1F, G). Transwell assays revealed that migration and invasion were elevated in the ADR-ZsGreen^+^ cells compared to the other two types of cells (Fig. 1H). The enhancement in proliferation, migration and invasion was also observed in the ZsGreen^+^ populations derived from BT474 and MDA-MB-231 cells recovered from treatment with cisplatin (CDDP), another widely-used chemotherapeutic drug in clinic (Fig. S1C–E). These data together suggest that breast cancer cells elevate proliferation, migration and invasion after chemotherapeutic drug-induced anastasis.

### Anastatic breast cancer cells exhibit enhanced growth and metastasis in vivo

**Fig. 2:**
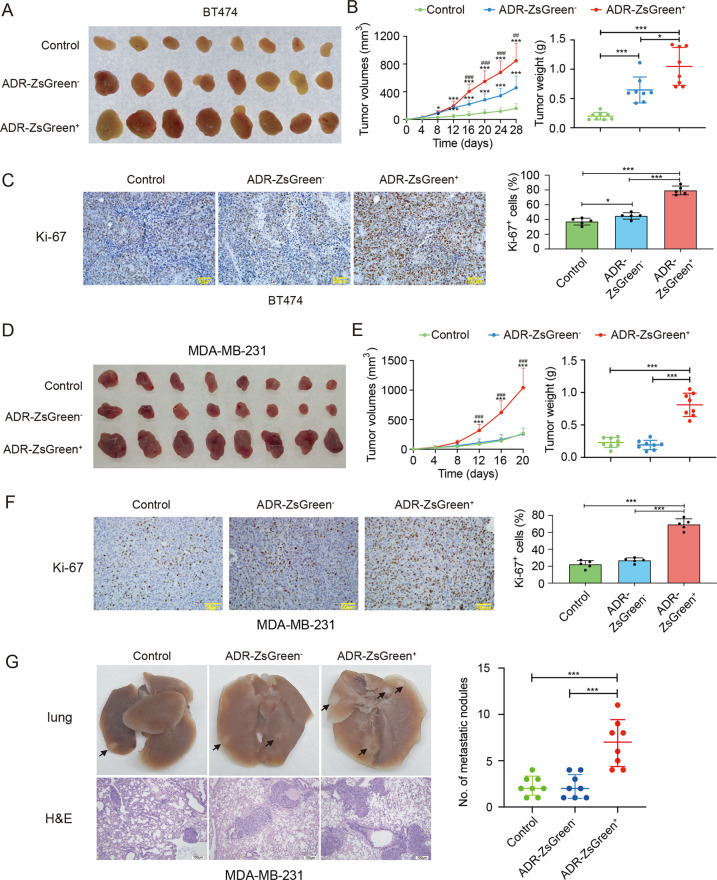
Anastasis promotes tumor growth and metastasis. **A** Image of xenografts formed by BT474-ADR-ZsGreen^+^, BT474-ADR-ZsGreen^−^ and BT474-control cells in nude mice. **B** On the left are the growth curves of the xenografts. * Indicates significant difference compared to the control group. # Indicates significant difference compared to the ADR-ZsGreen^−^ group. The bar graph on the right shows the weights of the xenografts in (**A**). *n* = 8. **C** Ki-67 expression in xenografts formed by the indicated cell populations derived from BT474 cells. Left: representative images of immunohistochemistry (IHC) staining. Scale bar, 50 μm. Right: quantification of the percentage of Ki-67^+^ cells in tumors. *n* = 5. **D** Image of xenografts formed by MDA-MB-231-ADR-ZsGreen^+^, MDA-MB-231-ADR-ZsGreen^−^ and MDA-MB231-control cells in nude mice. **E** On the left are the growth curves of the xenografts. * Indicates significant difference compared to the control group. # Indicates significant difference compared to the ADR-ZsGreen^−^ group. The bar graph on the right shows the weights of the xenografts in (**D**). *n* = 8. **F** Ki-67 expression in xenografts formed by the indicated cell populations derived from MDA-MB-231 cells. Left: representative images of IHC staining. Scale bar, 50 μm. Right: quantification of the percentage of Ki-67^+^ cells in tumors. *n* = 5. **G** Lung metastasis of MDA-MB-231-ADR-ZsGreen^+^, MDA-MB-231-ADR-ZsGreen^−^ and MDA-MB-231-control cells. Left: the representative images of the lungs and H & E staining. Black arrows in the upper row point to examples of tumor nodules. Scale bars in the lower row are 100 µm. Right: the number of metastatic tumor nodules in lungs from each mouse. *n* = 8. Data are presented as mean ± SEM. Statistical significance was determined using one-way ANOVA with Tukey test. * or # *P* < 0.05, ** or ## *P* < 0.01, *** or ### *P* < 0.001.

Next we evaluated the effect of anastasis on breast cancer malignancy in vivo. The ADR-ZsGreen^+^ cells, the ADR-ZsGreen^−^ cells and the control cells were injected into the mammary fat pads of nude mice to establish tumor xenograft mouse models. The xenografts formed by the ADR-ZsGreen^+^ cells grew faster and contained more Ki-67^+^ cells compared to the xenografts formed by the other two types of cells (Fig. 2A–F), suggesting that anastasis promotes in vivo tumor growth.

To determine whether anastasis influences metastasis, we injected the ADR-ZsGreen^+^, the ADR-ZsGreen^−^ and the control populations derived from MDA-MB-231 cells into nude mice through tail veins and assessed the formation of metastatic nodules in lungs. As shown in Fig. 2G, the ADR-ZsGreen^+^ cells produced significantly increased number of metastatic lung nodules than the other two cell populations, suggesting that anastasis contributes to breast cancer metastasis after chemotherapy.

### The enhanced malignancy in anastatic breast cancer cells relies on elevated CDH12 expression

**Fig. 3:**
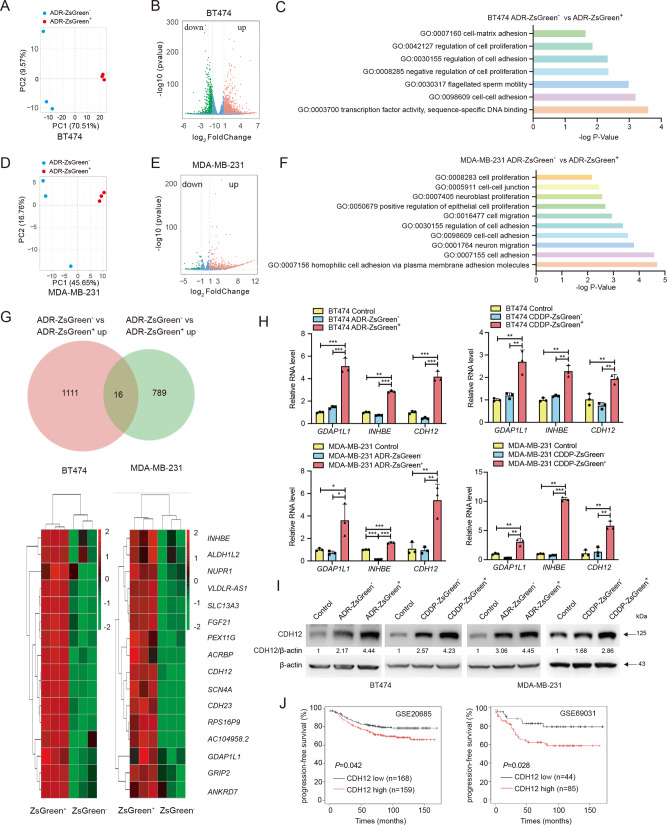
CDH12 is upregulated in anastatic breast cancer cells. **A** PCA of the RNA sequencing data of ADR-ZsGreen^+^ and ADR-ZsGreen^−^ cells derived from BT474 cells. **B** Volcano plot showing genes differentially expressed in BT474-ADR-ZsGreen^+^ and BT474-ADR-ZsGreen^−^ cells. **C** GO enrichment of genes upregulated in BT474-ADR-ZsGreen^+^ cells. **D** PCA of the RNA sequencing data of ADR-ZsGreen^+^ and ADR-ZsGreen^−^ cells derived from MDA-MB-231 cells. **E** Volcano plot showing genes differentially expressed in MDA-MB-231-ADR-ZsGreen^+^ and MDA-MB-231-ADR-ZsGreen^−^ cells. **F** GO enrichment of genes upregulated in MDA-MB-231-ADR-ZsGreen^+^ cells. **G** On the top is the Venn diagram showing genes that were commonly upregulated more than 4 folds in the ADR-ZsGreen^+^ cells derived from BT474 cells and MDA-MB-231 cells. The bottom is the heat map illustrating the expression level of the 16 commonly upregulated genes in each sample. **H** qRT-PCR results of the *GDAP1L1*, *INHBE* and *CDH12* in the ZsGreen^+^, the ZsGreen^−^ and the control cell populations derived from BT474 (top) or MDA-MB-231 (bottom) cells. *n* = 3. Data are presented as mean ± SEM. Statistical significance was determined using one-way ANOVA with Tukey test. *P < 0.05; **P < 0.01; ***P < 0.001. **I** Western blots of CDH12 in the indicated cell populations. **J** Kaplan–Meier plots showing that patients with high *CDH12* expression had significantly worse progression-free survival than those with low *CDH12* expression. Left: analysis result of GSE20685. Right: analysis result of GSE69031.

To investigate the molecular mechanism underlying the enhanced malignancy in anastatic breast cancer cells, we performed whole transcriptome sequencing on the ADR-ZsGreen^+^ and the ADR-ZsGreen^−^ populations derived from BT474 cells and MDA-MB-231 cells (Supplementary Dataset 1). Principal component analysis (PCA) and gene expressional analysis identified dramatical difference between the transcriptomes of the ADR-ZsGreen^+^ populations and those of the ADR-ZsGreen^−^ populations (Fig. 3A, B, D, E). Consistent with the elevated proliferation and migration in the ADR-ZsGreen^+^ populations, gene ontology (GO) analysis revealed that genes upregulated in the ADR-ZsGreen^+^ cells were enriched in GO terms related to cell proliferation, migration and adhesion (Fig. 3C, F). Given that proliferation and migration were enhanced in all the ZsGreen^+^ populations derived from BT474 and MDA-MB-231 cells after ADR or CDDP treatment, we looked for genes that were commonly upregulated in all the ZsGreen^+^ populations. Through analyzing the RNA sequencing data, we found that 16 genes were highly upregulated (more than 4 folds) in both the ADR-ZsGreen^+^ populations derived from BT474 cells and MDA-MB-231 cells compared to their ADR-ZsGreen^−^ counterparts (Fig. 3G). We assessed the expression of these 16 genes in all the ZsGreen^+^ and the ZsGreen^−^ populations generated from cells after ADR or CDDP treatments and the control cells. Only *GDAP1L1*, *INHBE* and *CDH12* were upregulated in all the ZsGreen^+^ cells (Fig. 3H, Fig. S2). Among them, *CDH12*, which encodes cadherin 12, has been linked to proliferation and invasion of colorectal cancer cells and salivary adenoid cystic carcinoma cells^23,24^. Consistent with the mRNA expression, CDH12 protein was upregulated in the ZsGreen^+^ cells compared to the ZsGreen^−^ cells and the control cells (Fig. 3I). Analysis of publicly available datasets revealed that breast cancer patients with high *CDH12* expression exhibited worse progression-free survival (Fig. 3J), implying the association of CDH12 with breast cancer malignancy.

**Fig. 4:**
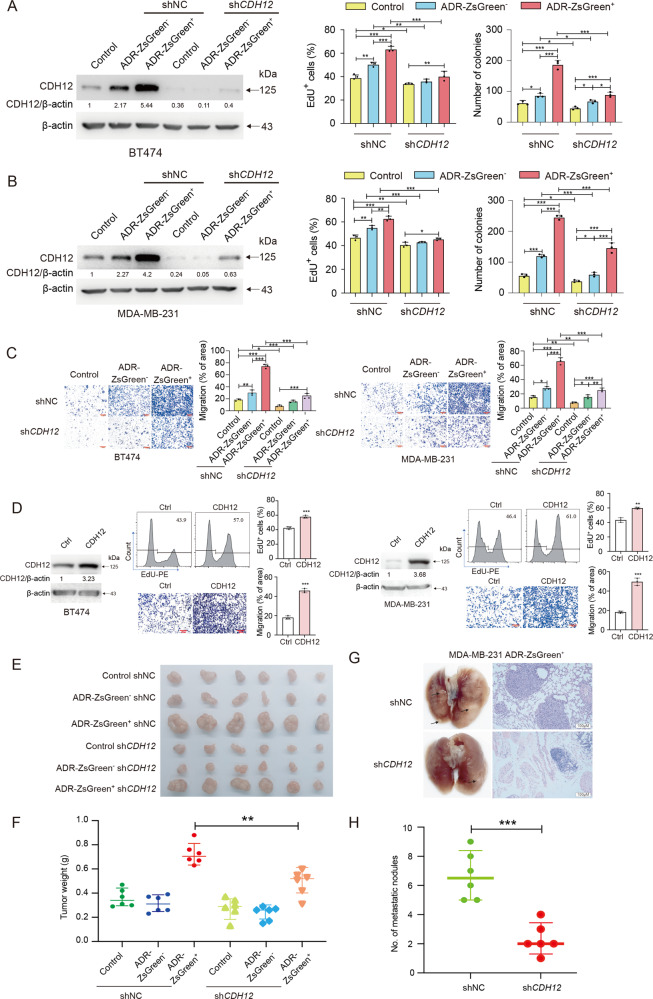
The elevated CDH12 promotes the enhanced malignancy in anastatic breast cancer cells. **A**, **B** The effect of *CDH12* knockdown in the indicated cell populations on EdU incorporation and colony formation. Left: Western blot showing the knockdown efficiency. Middle: the results of EdU incorporation assays. Right: the results of colony formation assays. *n* = 3. **C** The results of transwell migration assays on the indicated cell populations. Scale bars, 200 μm. *n* = 3. **D** The effect of CDH12 overexpression on EdU incorporation and transwell migration in BT474 and MDA-MB-231 cells. Scale bars, 200 μm. *n* = 3. **E** Image of the xenografts formed by the indicated cell populations derived from MDA-MB-231 cells. **F** The weights of the xenografts in (**E**). *n* = 6. **G**, **H** The effect of knocking down *CDH12* on lung metastasis of MDA-MB-231-ADR-ZsGreen^+^ cells. **G**: the representative images of lungs and H & E staining. Scale bars for the images of H & E staining are 100 μm. Black arrows point to examples of tumor nodules. **H**: the number of metastatic tumor nodules in lungs from each mouse. *n* = 6. Data are presented as mean ± SEM. Statistical significance was determined using one-way ANOVA with Tukey test for comparing three or more groups or t-test for comparing two groups. *P < 0.05; **P < 0.01; ***P < 0.001.

To determine the role of CDH12 in anastasis-induced enhancement in proliferation and migration, we delivered shRNA targeting *CDH12* (sh*CDH12*) into all the ZsGreen^+^, the ZsGreen^−^ and the control populations to generate cell lines with *CDH12* stably knocked down. Knocking down *CDH12* suppressed proliferation and migration in all cell populations and attenuated the difference among the ZsGreen^+^, the ZsGreen^−^ and the control populations (Fig. 4A–C, Fig. S3). Similar results were obtained in the ZsGreen^+^, the ZsGreen^−^ and the control cells transfected with a siRNA that targeted *CDH12* through a different sequence (Fig. S4). Moreover, overexpression of *CDH12* in the parental untreated BT474 or MDA-MB-231 cells dramatically increased cell proliferation and migration (Fig. 4D). These data suggest that the elevated proliferation and migration in anastatic breast cancer cells is due to upregulated CDH12.

We then assessed the effect of *CDH12* knockdown on in vivo tumor growth and metastasis. The volumes and weights of the xenografts formed by MDA-MB-231-ADR-ZsGreen^+^ cells with *CDH12* knocked down were significantly reduced compared with those formed by MDA-MB-231-ADR-ZsGreen^+^ cells expressing control shRNA (shNC), whereas xenografts formed by MDA-MB-231-ADR-ZsGreen^−^ cells and MDA-MB-231-control cells were only slightly affected by *CDH12* knockdown (Fig. 4E, F). In addition, reducing *CDH12* expression strongly suppressed lung metastasis of MDA-MB-231-ADR-ZsGreen^+^ cells (Fig. 4G, H). Taken together, these results indicate that increased expression of CDH12 is essential for the enhanced malignancy of breast cancer cells after anastasis induced by chemotherapeutic drugs.

### CDH12 promotes breast cancer malignancy via ERK-CREB

**Fig. 5:**
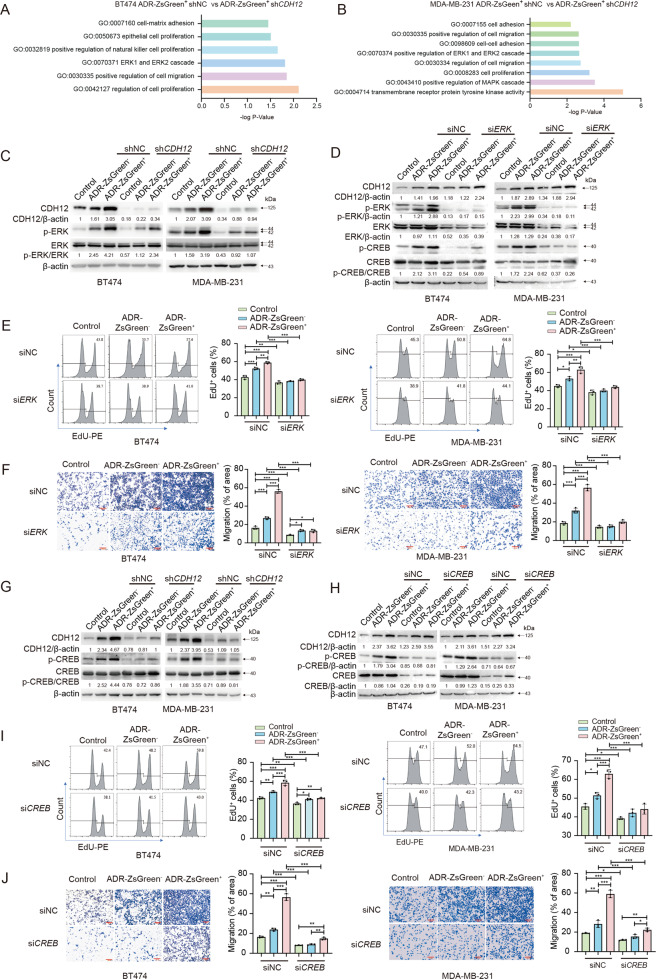
CDH12 promotes breast cancer cell proliferation and migration via ERK-CREB signaling. **A**, **B** GO enrichment analysis of genes downregulated after knocking down *CDH12*. **C** Western blots showing the effect of *CDH12* knockdown on the protein levels of CDH12, phosphorylated ERK (p-ERK) and ERK proteins. **D** Western blots showing the effect of *ERK* knockdown on CDH12, p-ERK, ERK, phosphorylated CREB (p-CREB) and CREB proteins. **E** The effect of *ERK* knockdown on EdU incorporation of the indicated cell populations. *n* = 3. **F** The effect of *ERK* knockdown on transwell migration of the indicated cell populations. *n* = 3. Scale bar: 200 μm. **G** Western blots showing the effect of knocking down *CDH12* on the protein levels of CDH12, p-CREB, CREB in the indicated cell populations. **H** Western blots showing the effect of *CREB* knockdown on the protein levels of CDH12, p-CREB, CREB. **I** The effect of *CREB* knockdown on EdU incorporation in the indicated cell populations. *n* = 3. **J** The effect of *CREB* knockdown on transwell migration of the indicated cell populations. *n* = 3. Scale bar: 200μm. Data are presented as mean ± SEM. Statistical significance was determined using one-way ANOVA with Tukey test. *P < 0.05; **P < 0.01; ***P < 0.001.

To elucidate the mechanism underlying CDH12 regulation of cell proliferation and migration, we performed RNA sequencing on ADR-ZsGreen^+^ cells expressing shNC or sh*CDH12* (Supplementary Dataset 1). GO enrichment analysis revealed that genes downregulated in *CDH12* knockdown cells were enriched in GO terms related to proliferation, adhesion, migration and ERK cascade (Fig. 5A, B). Consistently, Western blots showed that phosphorylation of ERK, which indicates ERK activation, increased with CDH12 in the ZsGreen^+^ cells and suppressed by knocking down *CDH12* (Fig. 5C, Fig. S5A, B). In addition, overexpression of *CDH12* in untreated BT474 cells or MDA-MB-231 cells elevated ERK phosphorylation (Fig. S6A). These data together suggest CDH12 positively regulates ERK activity. To determine whether ERK mediates CDH12 regulation of proliferation and migration, we treated *CDH12*-overexpressing cells with ERK specific inhibitor SCH772984. Inhibition of ERK activity prevented elevation of proliferation and migration in *CDH12*-overexpressing cells (Fig. S6A–C). Knocking down *ERK* using siRNA yielded similar results (Fig. S6D–F). Knocking down *ERK* also suppressed enhancement in proliferation and migration in anastatic breast cancer cells (Fig. 5D–F). These results together indicate CDH12 promotes proliferation and migration through activating ERK.

We next investigate what functions downstream of ERK. Previous study showed that CDH12 regulates neurite outgrowth through activation of CREB^25^. CREB has also been reported as a downstream effector of ERK^26^. Transcription factor enrichment analysis revealed that CREB binding sites were enriched in the promoter regions of genes upregulated in ADR-ZsGreen^+^ cells (Fig. S5C). Thus, we assessed the role of CREB in CDH12-ERK regulation of proliferation and migration. CREB phosphorylation was upregulated along with CDH12 and ERK phosphorylation in the ZsGreen^+^ cells and suppressed by knocking down *CDH12* or inhibiting ERK expression or activity (Fig. 5D, G, Fig. S5A, B, D). In addition, overexpression of *CDH12* in untreated BT474 or MDA-MB-231 cells elevated CREB phosphorylation, which was attenuated by inhibition of ERK activity or interfering ERK expression (Fig. S6A, D). These data suggest that CDH12 activates CREB through ERK. Importantly, inhibition of CREB activity by the specific inhibitor KG-501 or knocking down *CREB* reduced proliferation and migration of breast cancer cells and attenuated the difference between the ZsGreen^+^, the ZsGreen^−^ and the control populations (Fig. 5H–J, Fig, S5E–G). Interference of CREB activity or expression also suppressed increased proliferation and migration induced by *CDH12* overexpression (Fig. S6). These results together indicate that CDH12 promotes proliferation and migration of breast cancer cells through inducing CREB activation.

### *CDH12* is epigenetically de-repressed in anastatic breast cancer cells

**Fig. 6:**
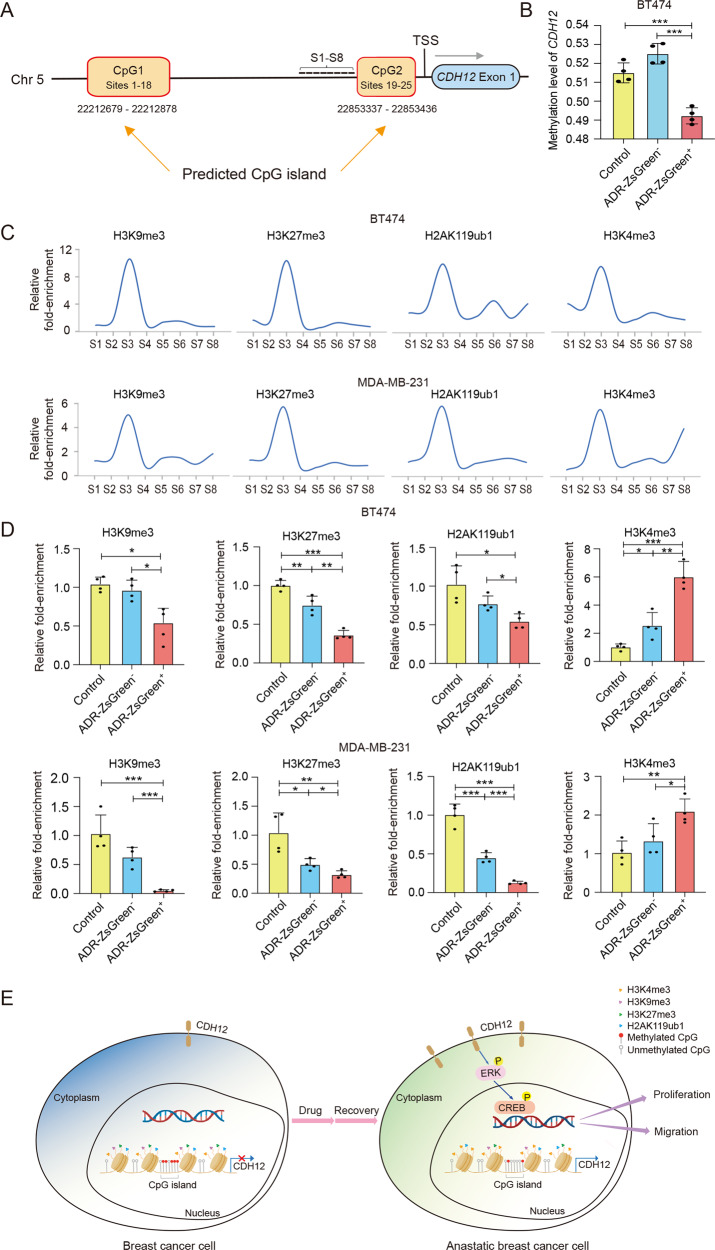
CDH12 is epigenetically de-repressed in anastatic breast cancer cells. **A** The schematic showing the CpG islands in the promoter region of *CDH12* and the positions targeted by primer sets S1–S8 (short lines) used in ChIP assays. **B** Methylation levels in *CDH12* promoter in BT474-ADR-ZsGreen^+^, BT474-ADR-ZsGreen^−^ and BT-474-control cells. *n* = 4. **C** Results of qChIP assays showing the enrichment patterns of the indicated histone modifications in *CDH12* promoter in BT474 and MDA-MB-231 cells. Results are represented as the fold-change over control (IgG). **D** Results of qChIP assays showing the enrichment of H3K9me3, H3K27me3, H2AK119ub1 and H3K4me3 in *CDH12* promoter in the indicated cell populations. *n* = 4. Data are presented as mean ± SEM. Statistical significance was determined using one-way ANOVA with Tukey test. *P < 0.05; **P < 0.01; ***P < 0.001. **E** Summary of the molecular mechanism underlying enhanced malignancy in anastatic breast cancer cells. In unstressed breast cancer cells, *CDH12* promoter is heavily methylated and enriched with repressive histone modifications, leading to low level of CDH12 protein. After exposure to chemotherapeutic drugs and survival from the induced executioner caspase activation, the DNA methylation and repressive histone modifications in *CDH12* promoter are removed, leading to upregulation in CDH12 proteins, which in turn promotes cancer cell proliferation and migration through activating ERK and CREB.

Given the persistently high expression of *CDH12* in anastatic cells, we wondered whether anastasis induces changes in epigenetic modifications at *CDH12* locus. We first investigated DNA methylation in the promoter region of *CDH12* gene. Two typical CpG islands were identified in the *CDH12* promoter region (Fig. 6A). Targeted bisulfite sequencing revealed that the methylation level on the two CpG islands in BT474-ADR-ZsGreen^+^ cells were significantly lower than that in BT474-ADR-ZsGreen^−^ cells and BT474-control cells (Fig. 6B). We then assessed the enrichment of repressive histone modifications like H3K27me3, H3K9me3 and H2AK119ub1 in the *CDH12* promoter region. Using a panel of 8 pairs of oligonucleotide primers (S1-S8) for a ~4.5 kb region (−4.5 kb to −0.1 kb) upstream of the transcription start site (TSS), we found that all these three modifications were enriched in the same region of *CDH12* promoter (Fig. 6C), and the enrichment was significantly reduced in the ZsGreen^+^ cells compared to the other two types of cells (Fig. 6D). In the meanwhile, the enrichment of the activating modification H3K4me3 was increased in the ZsGreen^+^ cells (Fig. 6D). Taken together, these results indicate that anastasis induces epigenetic alteration in the *CDH12* promoter region.

## Discussion

Chemotherapy, which kills cancer cells with cytotoxic drugs, is a commonly-used strategy for cancer treatment. However, some studies suggest that chemotherapy can promote tumor growth and spread, leading to tumor relapse and metastasis^27–29^. Previous studies have shown that chemotherapeutic drugs can modify tumor microenvironment through triggering the cytokine secretion from stromal cells, immune cells, or dying tumor cells^27–31^. In this study, we explored the role of anastasis, survival from stress-induced executioner caspase activation, in breast cancer relapse and metastasis occurring post-chemotherapy. Our data demonstrate that after exposure to chemotherapeutic drugs, breast cancer cells that survive from executioner caspase activation acquire enhanced proliferation and migration through de-repression of *CDH12* transcription and the subsequent activation of ERK/CREB signaling (Fig. 6E).

It has been reported that cervical cancer cells, ovarian cancer cells and melanoma cells that survive chemotherapeutic drug-induced executioner caspase activation acquire enhanced migration^13–15^. In this study, we also observed elevated migration and metastasis in the anastatic breast cancer cells after exposure to ADR or CDDP. Moreover, we found anastatic breast cancer cells were more proliferative, which has not been reported before. Although some studies have demonstrated that apoptotic cells or caspases can promote proliferation and migration both autonomously and non-autonomously^30,32–39^, anastatic cancer cells exhibit enhanced proliferation and migration after being separated from apoptotic cells and when they no longer have caspase activity, indicating neither apoptotic cells nor caspase activation is required for maintenance of the new features induced by anastasis. What drives the anastasis-induced phenotypic changes is an important question. Berthenet et al. have shown the requirement of JNK activation in the elevated migration in melanoma cells that survive executioner caspase activation induced by transient expression of tBid or exposure to dacarbazine^14^. Seervi et al. identified XPO1, a protein involved in nuclear export, essential for increased migration and drug resistance in anastatic cancer cells^13^. In this study, we show that upregulated expression of CDH12 and the subsequent activation of ERK/CREB signaling are responsible for the increased proliferation and migration of anastatic breast cancer cells. More importantly, we demonstrate that anastasis induces epigenetic alteration on *CDH12* promoter, which explains the persistently high level of CDH12 in the anastatic cells. Our previous RNA sequencing data on HeLa cells within the first 12 h recovery after removal of apoptotic stress revealed the enrichment of differentially expressed genes in GO term “chromatin modification”^19^. A recent work on colon cancer cells that survived from lethal treatment also found enrichment of downregulated genes in the processes “DNA methylation” and “PRC2 methylated histones”^31^, suggesting the involvement of epigenetic changes in cell recovery from severe stress. Basavarajappa group has reported that in the brains of mice exposed to ethanol, caspase-3 activation caused degradation of DNMT1, DNMT3A and MeCP, linking executioner caspase activation to changes in DNA methylation^40,41^. Given that the only difference between the ZsGreen^+^ cells and the ZsGreen^−^ cells is the experience of executioner caspase activation, the epigenetic alteration we observed in anastatic breast cancer cells may also attribute to the executioner caspase activation during drug treatment. Further efforts are needed to elucidate how executioner caspase activation causes epigenetic changes.

Cadherin proteins have been demonstrated to play important roles in carcinoma progression^42–45^. However, the studies regarding CDH12 are limited. CDH12 is highly expressed in tumor samples from patients with colorectal cancer or salivary adenoid cystic carcinoma and associated with metastasis and poor prognosis^23,24,46^. Recently, a study reported that patients with *CDH12*-enriched bladder cancers have poor outcome of neoadjuvant chemotherapy, and that *CDH12*-enriched cancer cells exhibit aggressiveness and chemoresistance^47^. This is consistent with our finding that elevated CDH12 expression drives enhanced malignancy in anastatic cancer cells after exposure to chemotherapeutic drugs.

Although CDH12 has been reported in colorectal cancer cells and salivary adenoid cystic carcinoma cells to promote proliferation, migration and epithelial mesenchymal transition^23,24,46^, little is known about the underlying molecular mechanism. CDH12 has been reported to regulate neurite outgrowth through cAMP-PKA-CREB signaling pathway^25^. CREB, a transcription factor frequently hyperactivated in a variety of cancers, can drive the malignant activities of cancer including proliferation, angiogenesis and metastasis^48–50^. We identified CREB as the mediator for cancer cell proliferation and migration driven by CDH12. However, in breast cancer cells, CDH12 activated ERK not PKA. Knocking down *CDH12* significantly suppressed ERK activation and inhibition of ERK blocked CDH12-induced CREB activation, indicating ERK mediates CDH12 regulation of CREB.

In conclusion, we demonstrate that anastasis after exposure to chemotherapeutic drugs induces de-repression of *CDH12* transcription, leading to enhanced malignancy in breast cancer cells. Our results unveil CDH12 as a positive regulator for post-chemotherapy relapse and metastasis of breast cancer.

## Materials and methods

### Cell culture

The breast cancer cell lines BT474 (Cat# TCHu143) and MDA-MB-231 (Cat# TCHu227) were obtained from National Collection of Authenticated Cell Cultures (Shanghai, China). BT474 cells were maintained in RPMI-1640 (Gibco, Grand Island, NY, USA, Cat# C11875500BT) complemented with 10% fetal bovine serum (FBS) (SuperCulture, Shenzhen, China, Cat# 60211031). MDA-MB-231 cells were maintained in DMEM (Gibco, Cat# C11995500BT) complemented with 10% FBS. All medium contained 100 U/ml penicillin and 100 μg/ml streptomycin (SparkJade, Shandong, China, Cat# CM0004). Cells were grown in a 5% CO_2_ incubator at 37 °C. All cells were routinely tested for mycoplasma. When treated or transfected, cells were randomly allocated into different treatment groups.

### Generation of stable cell lines

mCasExpress system contains two plasmids, pCDH-FRT-STOP-FRT-ZsGreen-puro plasmid and pCW57-Lyn11-NES-DEVD-flpO-hygro^15^. To generate stable *CDH12* knockdown and control cell lines, sh*CDH12* (TGGATTAGCCGGAACAACAATTCAAGAGATTGTTGTTCCGGCTAATCCTTTTTT) or shNC (TGTTCTCCGAACGTGTCACGTCAATTCAAGAGAACGTGACACGTTCGGAGAATTTTTT) was inserted into pLKO.1 vector. For stable lines overexpressing *CDH12*, pLVX-IRES-BSD-CDH12 were generated by sub-cloning the coding sequence of *CDH12* into the pLVX-IRES-BSD vector, and the empty pLVX-IRES-BSD was used as a control. Each of these plasmids was transfected into HEK293T cells together with pCMV-dR8.2 dvpr (Addgene# 8455) and pCMV-VSV-G (Addgene# 8454) using Lipofectamine 2000 (Invitrogen, New York, USA, Cat# 11668030). The supernatant was harvested and filtered with a 0.45 μm filter at 48 h post transfection and applied to breast cancer cells overnight in the presence of 10 μg/mL polybrene (Solarbio, Cat# H8761). The infected breast cancer cells were then selected for 7–10 days in growth medium containing 2 μg/mL puromycin (Solarbio, Beijing, China, Cat# P8230), 200 μg/mL hygromycin (Solarbio, Cat# H8081) or 10 μg/mL Blasticidin S (Solarbio, Cat# B9300).

### siRNA transfection

For the knockdown experiments, cells were transfected with si*CDH12* (sense: GCAAGCCACUUUACACCAUTT, antisense: AUGGUGUAAAGUGGCUUGCTT) or siNC (sense: UUCUCCGAACGUGUCACGUTT, antisense: ACGUGACACGUUCGGAGAATT)^25^, si*CREB* (Santa Cruz Biotechnology, Cat# sc-29281), or si*ERK* (Santa Cruz Biotechnology, Cat# sc-29307), using the Lipofectamine 2000 transfection reagent according to the manufacturer’s instructions.

### Isolation of the ZsGreen^+^, the ZsGreen^−^, and the control populations

BT474^Cas^ and MDA-MB-231^Cas^ cells were treated with 1 μg/mL DOX (Sangon Biotech, Shanghai, China, Cat# A600889) for 6 h. Then BT474^Cas^ cells were treated with 20 ng/mL ADR (MedChemExpress, Shanghai, China, Cat# HY-15142A) or 1 μg/mL CDDP (MedChemExpress, Cat# HY-17394) plus 1 μg/mL DOX. MDA-MB-231^Cas^ cells were treated with 30 ng/mL ADR or 3 μg/mL CDDP plus 1 μg/mL DOX. 24 h later, the treatment medium was replaced with fresh growth medium to allow cells to recovery. After 120 h recovery, the ZsGreen^+^ cells and ZsGreen^−^ cells were sorted on Beckman MoFlo (Beckman Coulter, Indianapolis, IN, US). To isolate control populations, cells were treated with 1 μg/mL DOX for 30 h, then grew in growth medium for 120 h and subjected to sorting on Beckman MoFlo.

### Colony formation assay

Cells were seeded at 1 × 10^3^ cells/well in six-well plates. After two weeks, the colonies were fixed with 4% paraformaldehyde and stained with 0.2% crystal violet (Solarbio, Cat# G1065). The colonies were counted and imaged on Olympus TH4–200 microscope (Olympus, Tokyo, Japan).

### EdU incorporation assay

EdU incorporation and staining were performed using EdU-555 kit (Beyotime, Shanghai, China, Cat# C0075) according to the manufacturer’s instructions. The percentage of EdU^+^ cells were measured using CytoFLEX S (Beckman, USA).

### Migration and invasion assay

Cell migration and invasion assays were performed using 24-well BD Falcon 8.0 μm transwell inserts (Falcon, BD Biosciences, MA, USA, Cat# 353097). For migration assay, 3 × 10^5^ BT474 cells or 5 × 10^4^ MDA-MB-231 cells were seeded into the top chamber of the insert. For invasion assay, 3 × 10^5^ BT474 cells or 5 × 10^4^ MDA-MB-231 cells were seeded into the top chamber of the insert coated with Matrigel (BD Biosciences, Billerica, MA, USA, Cat# 354234). The upper chamber was filled with serum-free medium, and the lower chamber was filled with the medium containing 10% FBS. After 48 h (for BT474 cells) or 12 h (for MDA-MB-231 cells) incubation, the migrated and invaded cells were fixed with 4% paraformaldehyde (Solarbio, Cat# P1110) and stained with 0.5% crystal violet. The migrated and invaded cells were imaged using an inverted microscope (IX73, Olympus, Tokyo, Japan), and the migration or invasion capacity was determined by the percentage of area covered by the migrated or invaded cells, which was quantified using Image J software (National Institute of Health, USA).

### RNA extraction and quantitative RT-PCR

Total RNA was extracted using TRIzol Reagent (Thermo Fisher Scientific, MA, USA, Cat# 15596026). mRNA was first converted into cDNA using RevertAid Reverse Transcriptase (Thermo Fisher Scientific, MA, USA, Cat# K1691) according to the manufacturer’s instructions. Quantitative RT-PCR (qRT-PCR) was performed using FastStart Essential DNA Green Master (Roche, Basel, Switzerland, Cat# 4368706). β-actin was used as an internal reference. Primers used are listed in Table S1.

### Western blot

Protein extraction was performed using RIPA buffer (Beyotime, Cat# KGP702) supplemented with 1 mM PMSF (Keygen, Nanjing, China, Cat# KGP610). Protein samples were quantified using the BCA assay kit (Spark Jade, Cat# EC0001). 40 μg protein was separated in SDS-PAGE and transferred onto PVDF membranes. Membranes were incubated overnight at 4 °C with primary antibodies (1:1000), and then with secondary antibodies (1:5000) at room temperature for 1 h. Protein bands were detected using Clarity Western ECL blotting substrates (Thermo, Cat# 32209) and ChemiDoc Imager (Tanon, 5200). The antibodies used are listed in Table S2.

### RNA sequencing

Total RNA of cells was extracted with TRIzol reagents (Thermo) following the manufacturer’s protocol. mRNA was enriched using Oligo(dT) beads, fragmented, reversely transcribed into cDNA, and ligated to Illumina sequencing adapters. The cDNA library was sequenced using Illumina nova 6000 by Novogene Biotechnology Co. (Beijing, China). Genes with fold change more than 2 and false detection rate less than 0.05 were considered differentially expressed. GO enrichment analysis was performed using DAVID (https://david.ncifcrf.gov). Transcription factor enrichment analysis was performed using ChEA3 (https://maayanlab.cloud/chea3/).

### Xenograft model

All animal studies were conducted following the protocol approved by the Animal Care and Use Committee of School of Basic Medical Sciences of Shandong University. Six-week-old female BALB/c nude mice (Beijing Vital River Laboratory Animal Technology, Beijing, China, Cat# 401) were randomly allocated into different groups. 5 × 10^6^ BT474 cells or 2 × 10^6^ MDA-MB-231 cells were inoculated into the mammary fat pads of the mice. Tumors were measured with a caliper, and the tumor volume was calculated using the formula V = maximal diameter × perpendicular diameter^2^/2. When the largest tumor reached the maximum allowed size, mice were sacrificed. The tumors were surgically removed, weighted, photographed. Portions of the tumors were immediately frozen in liquid nitrogen for preparation of RNA and protein samples or fixed in 4% buffered formalin for immunohistochemistry.

### Immunohistochemistry

Tumor sections were embedded in paraffin wax. After deparaffinization and rehydration, the sections were subjected to antigen recovery then immersed in 3% H_2_O_2_ for 10 min to quench endogenous peroxidase. After being blocked with 10% goat serum at room temperature for 1 h, the sections were incubated with anti-Ki-67 antibody (1:200, Cell Signaling Technology, Danvers, MA, USA, Cat# 9027 T) overnight at 4 °C and second antibody (1:200, Jackson ImmunoResearch, Philadelphia, PA, USA, Cat# 111-035-003) at 37 °C for 1 h. The immunoreactions were visualized using horseradish peroxidase (HRP) conjugated DAB. Negative controls were performed by omitting the primary antibodies. Sections were observed and imaged using Olympus BX51 microscope (Olympus, Japan).

### In vivo lung metastasis assay

2 × 10^6^ cells MDA-MB-231 cells were resuspended in 100 μL PBS and injected through the tail vein into BALB/c nude mice. After 6 weeks, the mice were sacrificed, and the lungs were collected, fixed and analyzed.

### Targeted bisulfite sequencing assay

Genomic DNA was extracted from cells with QIAGEN kit (QIAGEN, Hilden, Germany) according to the manufacturer’s protocol. DNA was quantified and diluted to 20 ng/μL. Genomic DNA (400 ng) was subjected to sodium bisulfite treatment using EZ DNA Methylation™-GOLD Kit (Zymo Research) according to manufacturer’s protocol. CpG islands adjacent to the promoter region of *CDH12* gene were assessed by Genesky BioTech (Shanghai, China). Briefly, CpG islands were selected for measurement according to the following criteria: 0.60 or higher ratio of observed/expected dinucleotides CpG; 50% or higher GC content; 100 bp minimum length. Two regions from CpG islands of *CDH12* were selected and sequenced. PCR amplicons of target CpG regions were separated by agarose electrophoresis and purified using QIAquick Gel Extraction kit (QIAGEN, Hilden, Germany, Cat# 28704). The products were sequenced on an Illumina MiSeq benchtop sequencer (Illumina, CA, United States).

### ChIP-qPCR assay

ChIP assays were performed as described previously^51^. The site-specific primers used in qPCR are listed in Table S3. The antibodies used in ChIP assays are listed in Table S4.

### Statistical analysis

All data are presented as the mean ± standard error of the mean (SEM). Data were analyzed using GraphPad Prism 5 (GraphPad software Inc., San Diego, CA, USA). One-way Analysis of Variance (ANOVA) with Tukey test was used for comparison of three or more groups. The two-tailed unpaired *t*-test was used to analyze the difference between two groups. *P* < 0.05 was considered statistically significant. The assumption of equal variance was validated by *F*-test. No data were excluded from analysis. The sample sizes were chosen empirically based on the observed effects and previous reports. When collecting and analyzing data of RT-qPCR, immunostaining and xenograft volumes, the investigators were blinded to the group allocation. All the experiments were repeated at least three times and the representatives were shown in the figures.

## Supplementary information

Supplementary information

## Data Availability

The raw data for RNAseq can be assessed at NCBI Bioproject with accession number PRJNA964783. All the other raw data supporting the findings of this study are available from the corresponding author upon request.
