# Supplementary material for: Chemotherapy-induced executioner caspase activation increases breast cancer malignancy through epigenetic de-repression of CDH12
Source: Oncogenesis. 2023 Jun 24;12(1):34. doi: 10.1038/s41389-023-00479-x (PMC10290709; doi:10.1038/s41389-023-00479-x)
Supplement: Supplementary file 1 — Supplementary information [file 41389_2023_479_MOESM1_ESM.pdf]

Supplementary information

Chemotherapy-induced executioner caspase activation increases breast cancer malignancy through epigenetic de-repression of *CDH12*

Wang et al.

This file contains the following contents.

Figure S1-S6 and their legends

Table S1-S4

Legend for Supplementary Dataset 1

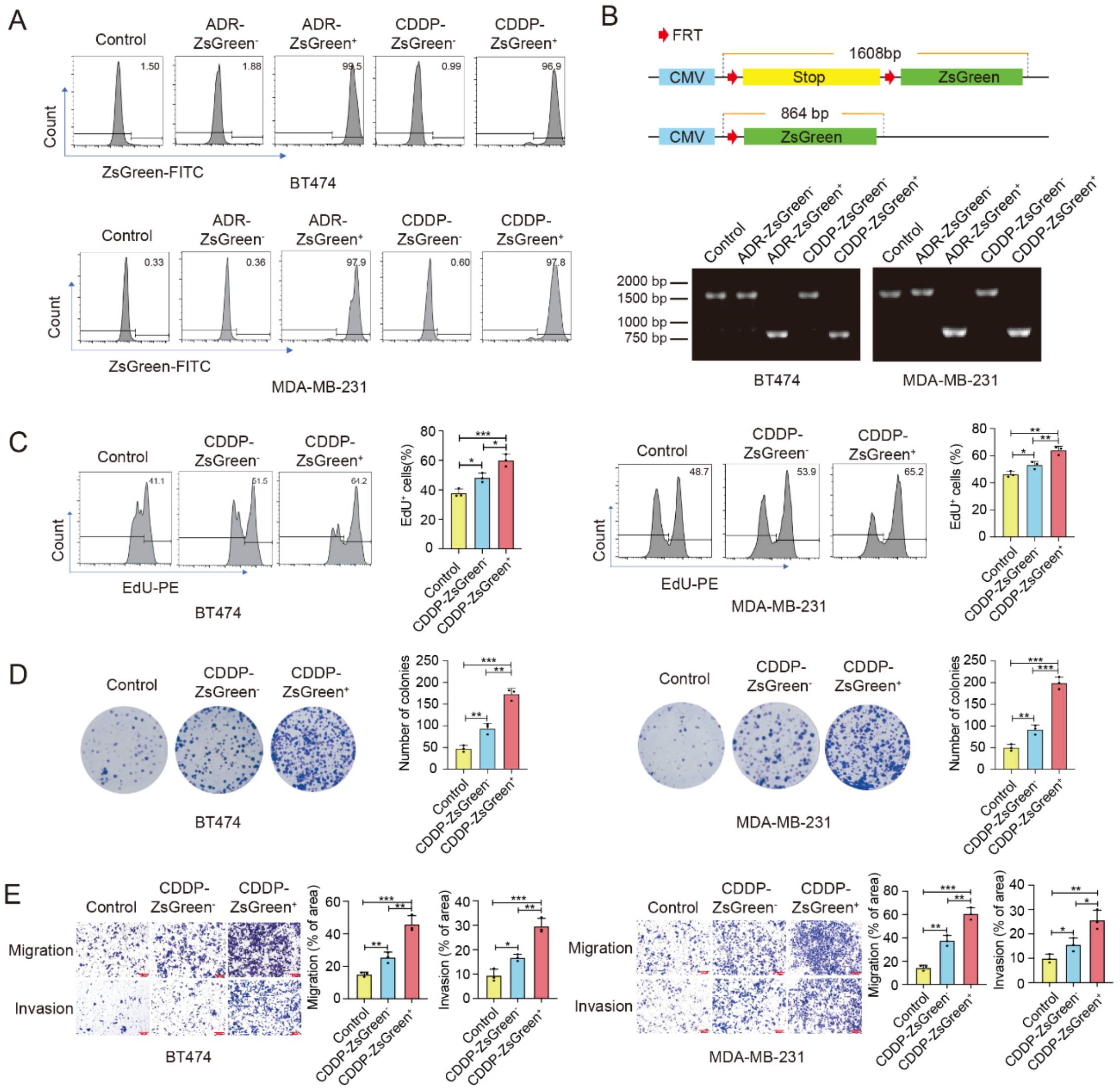

Figure S1. Anastatic breast cancer cells acquire enhanced proliferation and migration. A) Flow cytometry analysis showing the purity of the ZsGreen$^+$, the ZsGreen$^-$ and the control cell populations derived from BT474 (top) and MDA-MB-231 (bottom) cells. B) On the top is the schematic showing the positions of the genotyping primers (black dashlines) and the size of the PCR products. The bottom is the genotyping results of all the ZsGreen$^+$, the ZsGreen$^-$ and the control cell populations. C-E) The results of EdU incorporation analysis (C), colony formation assays (D) and transwell migration assays (E) on the CDDP-ZsGreen$^+$, the CDDP-ZsGreen$^-$ and the control cell populations derived from BT474 and MDA-MB-231 cells. n=3. Scale bars

in (E) are 200 μm. Data are presented as mean ± SEM. Statistical significance was determined using one-way ANOVA with Tukey test. * $P< 0.05$; ** $P< 0.01$; *** $P< 0.001$.

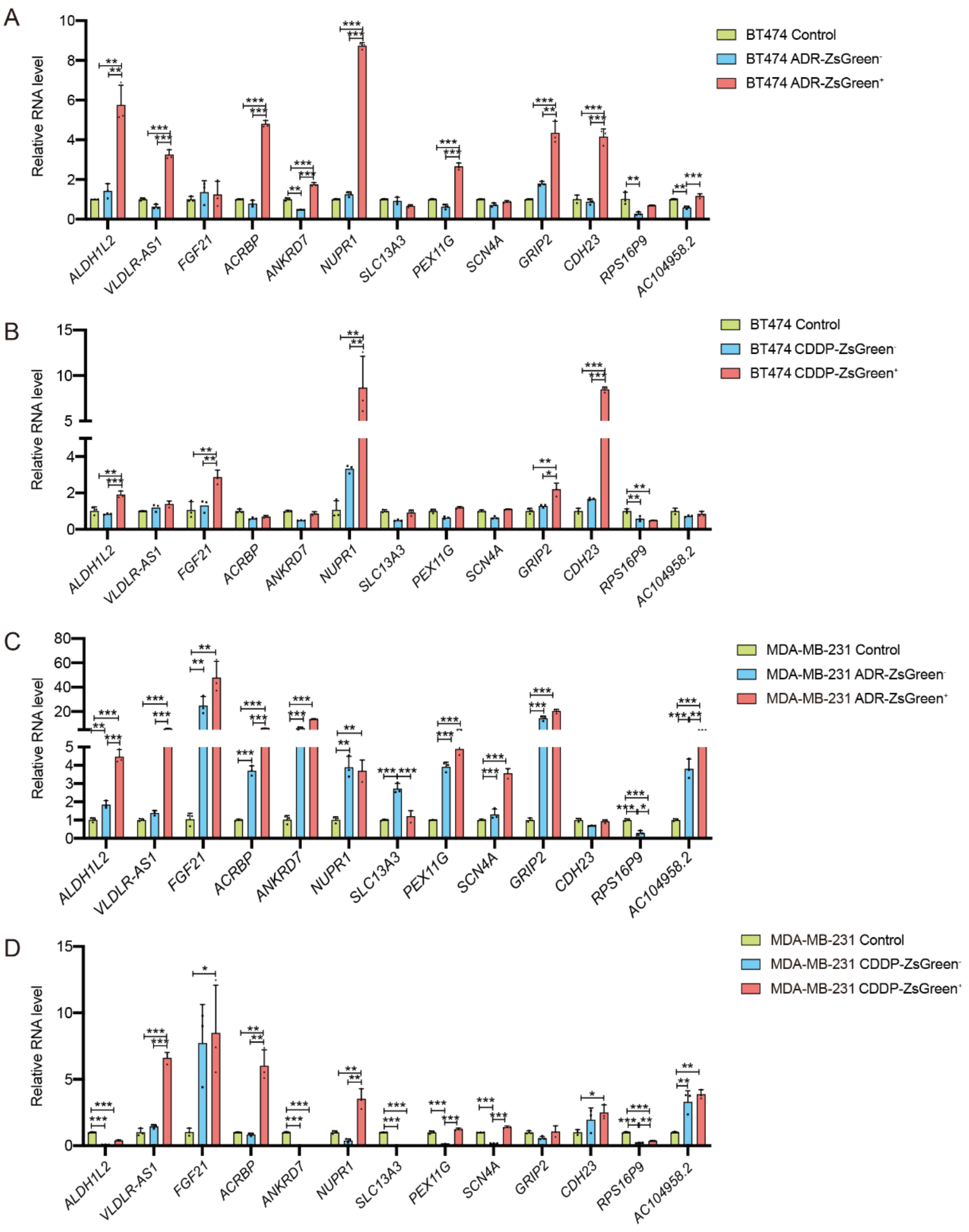

Figure S2. qRT-PCR validation of the commonly upregulated genes identified through RNA sequencing. A & B) qRT-PCR results of the indicated genes in the ZsGreen+ and the ZsGreen- populations derived from BT474 cells recovered from ADR (A) or CDDP (B) treatment and the control populations. C & D) qRT-PCR results of the indicated genes in the ZsGreen+ and the ZsGreen- populations derived from MDA-MB-231 cells recovered from ADR (C) or CDDP (D) treatment and the control

populations. n=3. Data are presented as mean ± SEM. Statistical significance was determined using one-way ANOVA with Tukey test. * $P< 0.05$; ** $P< 0.01$; *** $P< 0.001$.

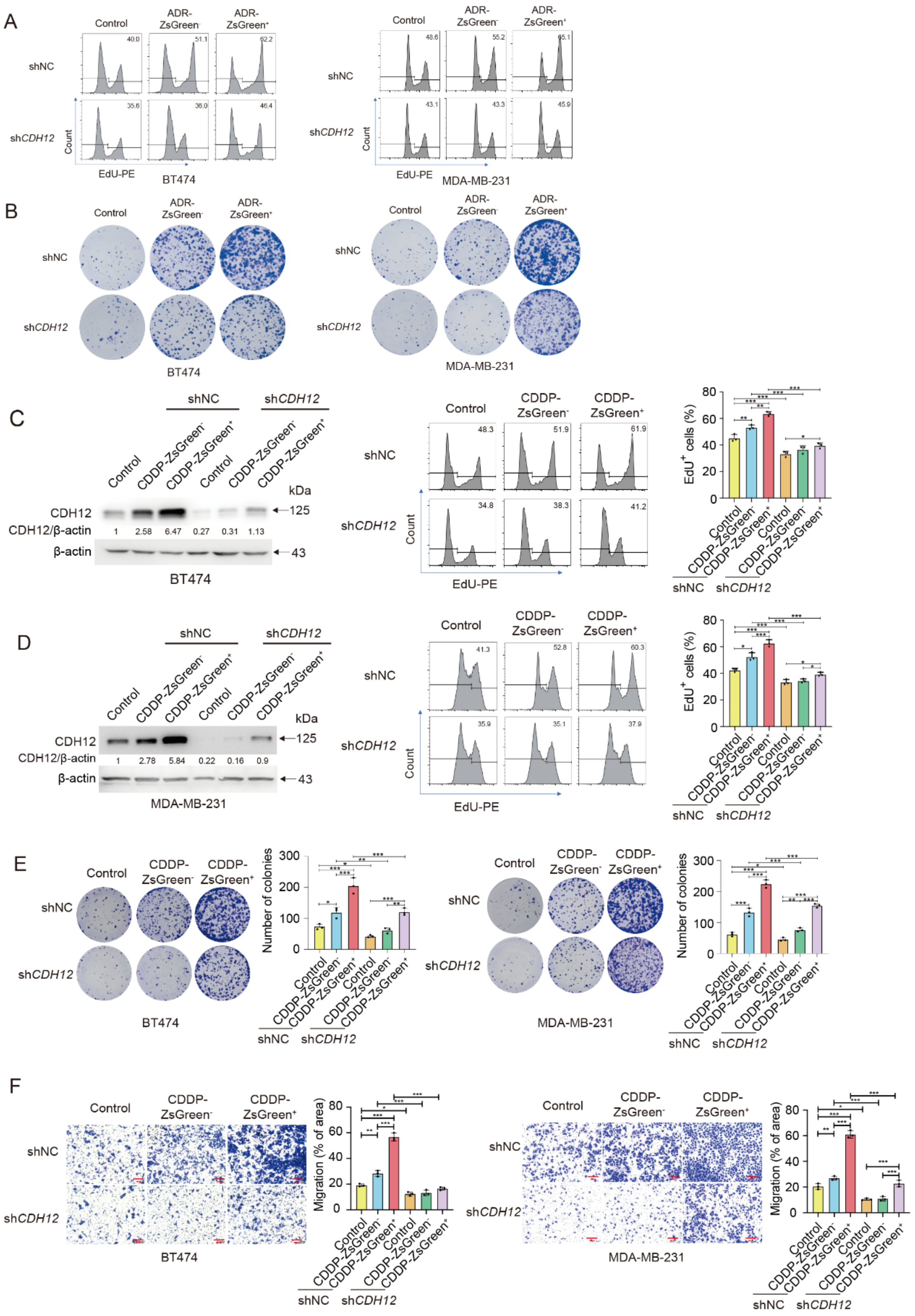

Figure S3. The enhanced proliferation and migration of the anastatic breast cancer cells rely on upregulated CDH12. A & B) The representative images for the bar graphs in Figure 4A & 4B. C & D) The results of EdU incorporation analysis to

show the effect of *CDH12* knockdown on the proliferation of the CDDP-ZsGreen$^+$, the CDDP-ZsGreen$^-$ and the control cell populations derived from BT474 (C) and MDA-MB-231 (D) cells. n=3. The Western blots on the left show the knockdown efficiency. E) The effect of *CDH12* knockdown on colony formation of the CDDP-ZsGreen$^+$, the CDDP-ZsGreen$^-$ and the control cell populations derived from BT474 and MDA-MB-231 cells. n=3. F) Transwell assays showing the effect of *CDH12* knockdown on migration of the CDDP-ZsGreen$^+$, the CDDP-ZsGreen$^-$ and the control cell populations derived from BT474 and MDA-MB-231 cells. n=3. Scale bar, 200 μm. Data are presented as mean ± SEM. Statistical significance was determined using one-way ANOVA with Tukey test. * $P < 0.05$; ** $P < 0.01$; *** $P < 0.001$.

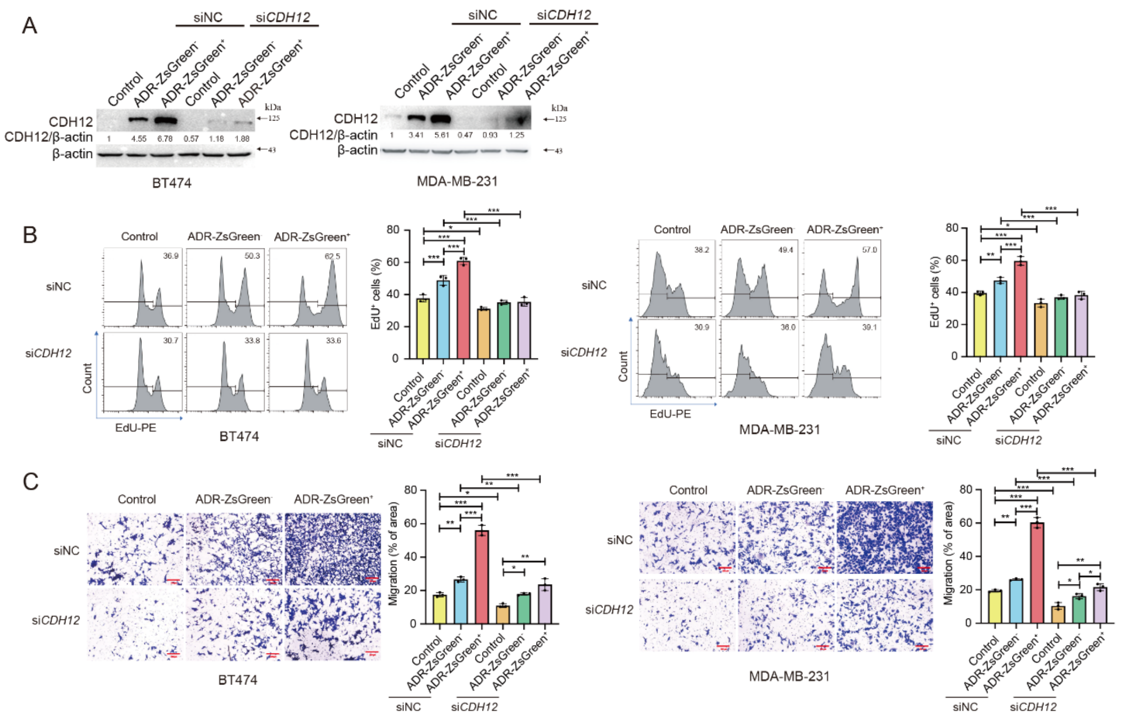

Figure S4. Interference of *CDH12* expression suppresses anastasis-induced enhancement in proliferation and migration. A) Western blots showing the knockdown efficiency of si*CDH12*. B) EdU incorporation assays to assess the effect of knocking down *CDH12* on proliferation of the indicated cell populations. n=3. C) Transwell assays to determine the effect of knocking down *CDH12* on migration of the indicated cell populations. n=3. Scale bar, 200 μm. Data are presented as mean ± SEM. Statistical significance was determined using one-way ANOVA with Tukey test. * $P< 0.05$; ** $P< 0.01$; *** $P< 0.001$.

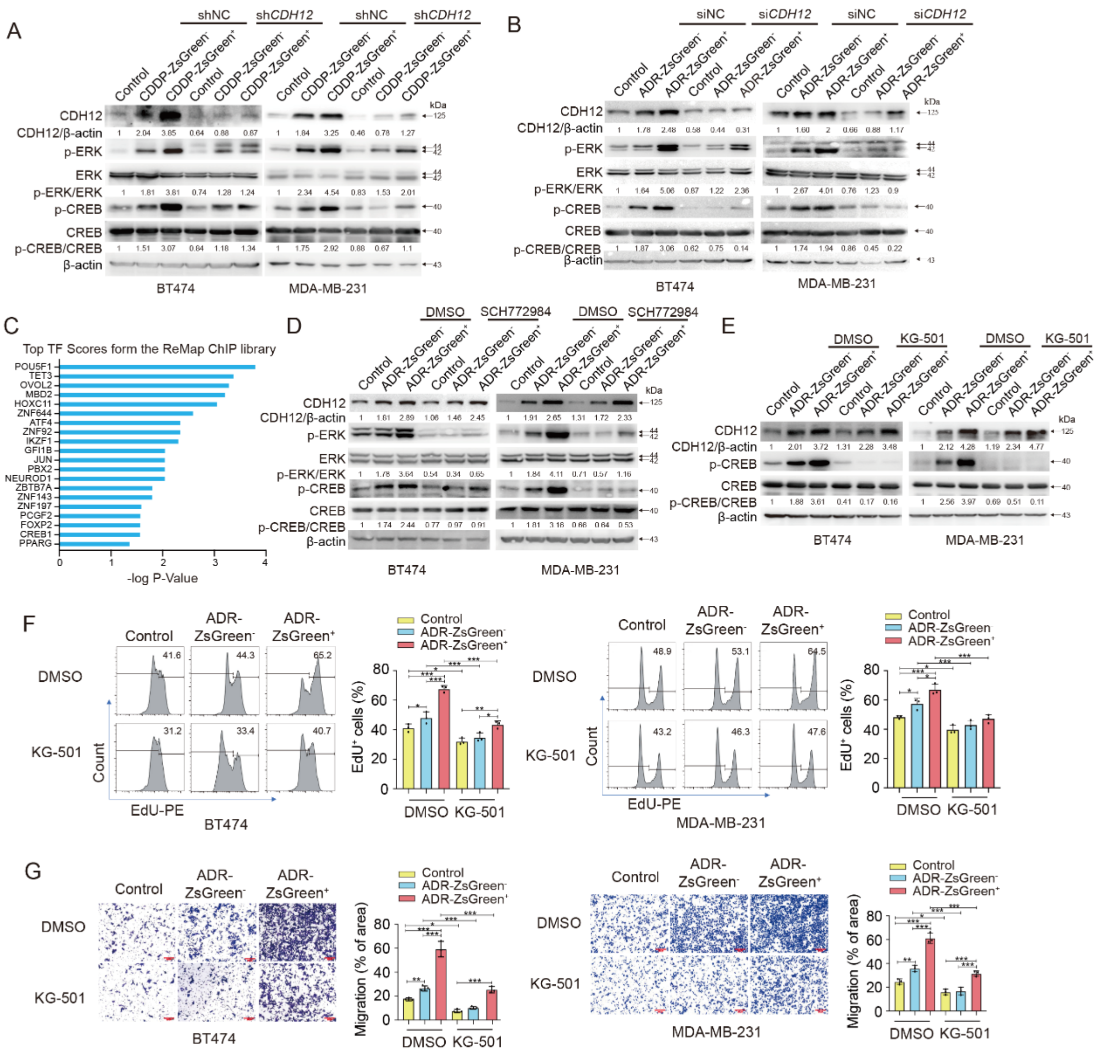

Figure S5. CDH12 promotes proliferation and migration of breast cancer cells through ERK-CREB. A & B) Western blots showing the effect of *CDH12* knockdown with shRNA (A) or siRNA (B) on CDH12, p-ERK, ERK, p-CREB and CREB proteins in the indicated cell populations. C) Transcription factor enrichment analysis of genes upregulated in ADR-ZsGreen^{+} cells identified through RNA sequencing. D) Western blots showing the effect of ERK inhibitor SCH772984 on CDH12, p-ERK, ERK, p-CREB and CREB proteins in the indicated cell populations. E) Western blots showing the effect of CREB inhibitor KG-501 on CDH12, p-CREB and CREB proteins in the indicated cell populations. F) The effect of KG-501 on EdU incorporation in the indicated cell populations. n=3. G) The effect of KG-501 on transwell migration of the

indicated cell populations. n=3. Scale bar, 200 μm. Data are presented as mean ± SEM. Statistical significance was determined using one-way ANOVA with Tukey test. * $P < 0.05$; ** $P < 0.01$; *** $P < 0.001$.

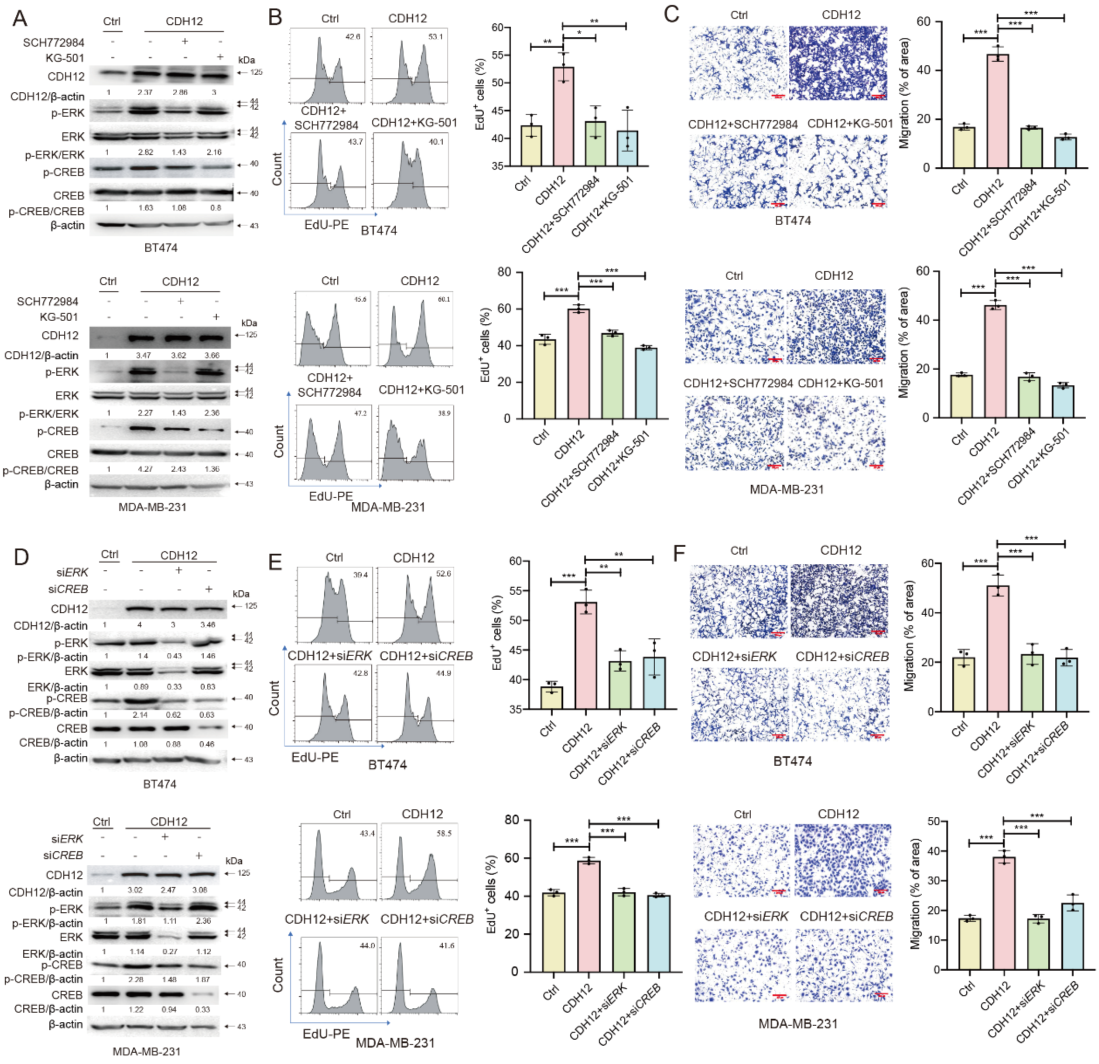

Figure S6. Overexpression of *CDH12* in breast cancer cells promotes proliferation and migration through activating ERK-CREB. A) Western blots showing the effect of treatment with ERK inhibitor SCH772984 or CREB inhibitor KG-501 on the protein levels of CDH12, p-ERK, ERK, p-CREB and CREB in the *CDH12*-overexpressing cells. B) The effect of ERK inhibitor SCH772984 or CREB inhibitor KG-501 on the elevated proliferation induced by *CDH12* overexpression. n=3. C) The effect of ERK inhibitor SCH772984 or CREB inhibitor KG-501 on the elevated migration induced by *CDH12* overexpression. n=3. Scale bar, 200 μm. D) Western blots showing the effect of knocking down *ERK* or *CREB* on the protein levels of CDH12, p-ERK, ERK, p-CREB and CREB in the *CDH12*-overexpressing cells. E) The effect of knocking down

ERK or *CREB* on the elevated proliferation induced by *CDH12* overexpression. n=3. F) The effect of knocking down *ERK* or *CREB* on the elevated migration induced by *CDH12* overexpression. n=3. Scale bar, 200 μm. Data are presented as mean ± SEM. Statistical significance was determined using one-way ANOVA with Tukey test. * $P < 0.05$; ** $P < 0.01$; *** $P < 0.001$.

Table S1. Primer sequences used for qRT-PCR

Primer name	Primer sequence (5'-3')
β-actin F	TGACGGGGTCACCCACACTGTGCCCATCTA
β-actin R	CTAGAAGCATTTGCGGTGGACGATGGAGGG
INHBE F	CTGTGACTGGAGGCATCAGA
INHBE R	CTCCCTCCCTAGTCCCTGAC
ALDH1L2 F	ACAAGGAAAAGGGGTGCTTT
ALDH1L2 R	CTATGGTCCAAACGCCTTGT
NUPR1 F	GGCAAACAGGAAGTGTGGTT
NUPR1 R	AAGTGGAAGCCATGTTTTGG
VLDLR-AS1 F	AGGAGGAAGAAGGCTCCAAG
VLDLR-AS1 R	TTCTTTCTTGCCTTGCTGGT
SLC13A3 F	AGGGGCTGGAGGAAGAATAA
SLC13A3 R	CGGTGACAGCATCAGAAAGA
FGF21 F	TGCCACGATGGAATTCTGTA
FGF21 R	TCGGAGAAGCTGAGTGGTTT
PEX11G F	CTGGTTGGTGGAGTTCTGGT
PEX11G R	TAGAGCTGGTCAGCCAGGTT
ACRBP F	CCACATGGACTTCTGGTGTG
ACRBP R	CTGCTGGCTTTTGAAGGAAC
SCN4A F	TTCACAGGCATCTTCACAGC
SCN4A R	GGAAGGAGCGTAGCACAGAC
GDAP1L1 F	CTCTGAATGGGGCTGGATAA
GDAP1L1 R	ACCCAACACATGGACCAAAT
GRIP2 F	AGCCCAAGGCTAAAGAGGAG
GRIP2 R	AGATCTGGCAGCAGTCACCT
ANKRD7 F	GAGAAAGGGGCTGATGTGAA
ANKRD7 R	CTCGCAGTGAATTGTGGGTA

CDH23 F	CCTGGTGTTTGGCGTGTCT
CDH23 R	GGCTGATTGTGAAATGTGGG
CDH12 F	TTTCTACACTCTTCGTGCTCAGG
CDH12 R	TGTTTCCATAGGTCGGGTCA
RPS16P9 F	TGCTGTCAGTCCATCTCCAG
RPS16P9 R	TGATCGTCATGATGGGCTTA
AC104958.2-F	TCAGTTACGTCCCCTTCCCA
AC104958.2-R	CCTTGCTGGGTTGGGTTTAC

Table S2. List of antibodies used in Western blotting

Name	Manufacturer & Catalog Number
CDH12	Abcam (Cat# ab181860)
CREB	Proteintech (Cat# 12208-1-AP)
p-CREB	Abcam (Cat# ab32096)
ERK	Cell Signaling Technology (Cat# 4695)
p-ERK	Cell Signaling Technology (Cat# 4370)
β-actin	Santa Cruz Biotechnology (Cat# sc-8432)
Second antibody	Jackson ImmunoResearch (Cat# 111-035-003)

Table S3. Primer sequences used for qChIP assay

Primer name	Primer sequences (5'-3')
S1 (-4389 ~ -4495)	F: ATGCCAAAATACTCAACATCACAG
	R: GACTCTGTCTCAAAGGAAAAAAAAA
S2 (-3707 ~ -3820)	F: TTTCTGTTTTTTTTGCTTGGGA
	R: GTAAAGGGGCTTCATTGAGGTG
S3 (-3126 ~ -3376)	F: TTTTGGGGTCAGAAGAGAATGT
	R: CAAAGGGGAGGGAAAGAAGATAT
S4 (-2695 ~ -2850)	F: ATGGAACTTGACTCACTGGATGTA
	R: TGCTATAAGATTACTCTGTTGGCG
S5 (-2025 ~ -2144)	F: CTACAGGGAGAGCAGCAAAAAT
	R: AGGTGGAGTGGAGAAGATCAAA
S6 (-1634 ~ -1836)	F: TACATAAAGAAATCGGCACCACT
	R: CCCAAGCCTATCAAATCAGAAAC
S7 (-939 ~ -1170)	F: CTACACCACCTACATCAACACATTT
	R: TCAGACATCAAGAATGCCACAA
S8 (-163 ~ -276)	F: GCCTAATCTTCACCCGCTG
	R: TGTTTGTTCTTTTTCCCCCCTA

Table S4. Antibodies used in ChIP assays

Name	Manufacturer & Catalog Number
H2AK119ub1	Cell Signaling Technology (Cat# 8240)
H3K27me3	Cell Signaling Technology (Cat# 9733)
H3K9me3	Cell Signaling Technology (Cat# 4658)
H3K4me3	Cell Signaling Technology (Cat# 9751)

Supplementary Dataset 1. List of differentially expressed genes identified in RNA sequencing.

This Excel file contains list of differentially expressed genes from comparison between BT474-ADR-ZsGreen^{-} and BT474-ADR-ZsGreen^{+}, comparison between MDA-MB-231-ADR-ZsGreen^{-} and MDA-MB-231-ADR-ZsGreen^{+}, comparison between BT474-ADR-ZsGreen^{+}-shNC and BT474-ADR-ZsGreen^{+}-sh*CDH12*, and comparison between MDA-MB-231-ADR-ZsGreen^{+}-shNC and MDA-MB-231-ADR-ZsGreen^{+}-sh*CDH12*.
